# Monteggia-like lesions – treatment strategies and one-year results

**DOI:** 10.3205/iprs000072

**Published:** 2015-12-15

**Authors:** Reinhold Laun, Michael Wild, Lars Brosius, Mohssen Hakimi

**Affiliations:** 1 Vivantes Klinikum Neukölln, Department of Orthopedic and Trauma Surgery, Berlin, Germany; 2 Klinikum Darmstadt, Department of Orthopedic, Trauma and Hand Surgery, Darmstadt, Germany; 3 Vivantes Klinikum Am Urban, Department of Orthopedic, Trauma and Hand Surgery, Berlin, Germany

**Keywords:** Monteggia-like lesion, Monteggia fracture, Monteggia equivalent

## Abstract

**Introduction: **The eponym “Monteggia fracture” includes various patterns of complex fracture-dislocations of the proximal ulna and radius, which are not well defined yet. They are frequently described as Monteggia-like lesions or Monteggia equivalent injuries. Until today, these injury patterns have been reported rarely. The objective of this retrospective study was to better define patterns of injury and to document the short-term results of treatment with current fixation techniques.

**Methods: **Ten patients with a Monteggia-like lesion were included in this study and clinical and radiological follow-up examinations at an average of 12.3 months after the trauma were performed. For clinical follow-up the Mayo Modified Wrist Score, the Mayo Elbow Performance Score, the functional rating index of Broberg and Morrey, and the DASH score were utilized.

**Results: **Osteosynthesis of the ulna was performed using a proximally contoured or precontoured LCP (locking compression plate) in all patients. All patients had a fracture of the radial head. All patients with a Mason type III radial head fracture received a cemented bipolar radial head prosthesis. All Mason type II fractures were treated with open reduction and internal fixation using mini screws. In all Mason type I fractures the treatment of the radial head dislocation was by closed reduction. Associated coronoid fractures were stabilized with lag screws through the ulnar plate or with independent lag screws after reduction of the fracture. According to the aforementioned scoring systems good to excellent results could be achieved.

**Conclusions: **Our findings demonstrate that good or excellent short-term results can be obtained if the injury is classified correctly and a standardized surgical treatment of all components of the injury is achieved. Further studies with larger patient populations and longer follow up periods are needed to evaluate long-term effectiveness of this treatment concept.

## Zusammenfassung

**Einleitung: **Der Begriff „Monteggia-Fraktur“ beinhaltet verschiedene Fraktur- und Verletzungskonstellationen an der proximalen Ulna und des Radius, welche bislang nicht eindeutig definiert wurden. Diese werden häufig als Monteggia-like lesions oder Monteggia-Äquivalent-Verletzungen beschrieben. Diese Verletzungsmuster wurden bis heute nur selten evaluiert. Das Ziel dieser retrospektiven Studie war die Erfassung von Ein-Jahres-Ergebnissen dieser seltenen Verletzungen nach deren Behandlung mit modernen Osteosyntheseverfahren.

**Methoden: **Zehn Patienten mit einer Monteggia-like lesion konnten in die Studie eingeschlossen und durchschnittlich nach 12,3 Monaten posttraumatisch klinisch und radiologisch nachuntersucht werden. Für die Nachuntersuchung wurden der Mayo Modified Wrist Score, der Mayo Elbow Performance Score, der Functional Rating Index von Broberg and Morrey und der DASH Score erhoben.

**Ergebnisse: **Bei allen Patienten erfolgte die osteosynthetische Versorgung der Ulna mittels einer proximalen intraoperativ anmodellierten oder bereits vorgeformten LCP (Locking Compression Plate). Alle Patienten wiesen eine Radiuskopffraktur auf. Alle Patienten mit einer Mason Typ III Radiuskopffraktur erhielten eine zementierte bipolare Radiuskopfprothese. Alle Mason Typ II Frakturen wurden mittels offener Reposition und Minischraubenosteosynthese operativ versorgt. Bei allen Typ I Frakturen erfolgte eine geschlossene Reposition. Begleitverletzungen des Processus coronoideus wurden mittels Zugschraubenosteosynthese behandelt. Bezüglich der ausgewählten Scoring Systeme konnten gute bis exzellente Ergebnisse erzielt werden.

**Schlussfolgerung: **Unsere Ergebnisse demonstrieren, dass nach korrekter Klassifikation und einer standardisierten operativen Behandlungsstrategie gute bis exzellente Ein-Jahres-Ergebnisse erzielt werden können. Weiterführende Studien mit größerem Patientenkollektiv und längerem Nachuntersuchungszeitraum sollten sich zur weiteren Evaluation dieses Behandlungskonzepts anschließen.

## Introduction

Monteggia fractures are rare injuries of the forearm and were first described by Giovanni Battista Monteggia in 1814 as a fracture of the shaft of the ulna combined with an anterior dislocation of the radial head [1]. Monteggia injuries account for only 2–5% of all proximal forearm fractures [2]. In 1967, Bado introduced the concept of a Monteggia lesion and presented a classification into four types depending on the direction of the radial head’s dislocation and the angulation of the fracture of the ulna [3], [4]. Type I describes a dislocation of the radial head in anterior, type II in posterior, and type III in lateral direction. Type IV is defined as a fracture of both bones of the forearm with dislocation of the radial head [4]. Furthermore, Bado described Monteggia equivalent injuries which differed in radiological appearance but possessed similar characteristics especially in the mechanism of injury and in its treatment. Type II injuries are most common (about 80%), followed by type I (about 15%), while type III and IV injuries are rare (all together 5%) [2]. Jupiter further classified the posterior Monteggia lesion (Bado type II) depending on the location and type of the ulnar fracture as well as the pattern of radial head injury [5], [6]. In type IIA, the fracture of the ulna involves the distal part of the olecranon and the coronoid process; in type IIB, the fracture involves the transition from metaphysis to diaphysis, distally to the coronoid process; in type IIC, a diaphyseal fracture is present and in type IID, the fracture extends from the olecranon to the proximal half of the ulna. Radial head fractures were classified in 4 types: type 0: no fracture; type 1: one part fracture; type 2: two part fracture; type 3: three part fracture; type C: comminuted fracture [4], [6].

The Bado and Jupiter classifications have been applied more frequently in the literature due to considerations of surgical practicality [3]. However, over the years additional injuries, such as radial head fractures, coronoid fractures, and combined radial head and coronoid fractures have added complexity to the classification of Monteggia-related injuries [3]. Not all of those injuries are included in the Bado and Jupiter classifications. Therefore the eponym of Monteggia fracture includes various patterns of complex fracture-dislocations of the proximal ulna and radius which are not well defined yet [6]. Other authors describe Monteggia lesions which include additional injuries such as radial head fractures (Mason, grade I-III) and coronoid fractures (Regan and Morrey I-III) as Monteggia-like lesions or Monteggia-equivalent injuries [2], [6], [7]. Nevertheless, according to Giannicola et al., in Monteggia-like pattern, six essential lesions can be identified and each of them must be recognized and treated:

1. Ulnar fracture,
2. Radio-humeral dislocation,
3. Ulnohumeral dislocation,
4. Proximal radio-ulnar dislocation,
5. Radial fracture and
6. Distal radio-ulnar joint\interosseus membrane lesion.

The various combination of these critical lesions can explain the complexity and variety of their treatment [6].

However, despite these confusing classifications and descriptions: Monteggia lesions with concomitant injuries to the radial head and/or the coronoid process do exist. Therefore, they should be considered separately.

We were able to diagnose and treat a total of 10 adult patients with Monteggia-like lesions which could be evaluated clinically and radiologically one year after the injury on average. The objective of this retrospective study was to better define circumstances and patterns of injury and to document the short-term results of treatment with current fixation techniques.

## Materials and methods

Ten adult patients were surgically treated for a Monteggia-like lesion between 2012 and 2014. Preoperatively, all patients received a CT-Scan of the injured elbow joint in order to rule out associated injuries and improve preoperative classification and planning. We defined a Monteggia-like lesion as a fracture of the proximal ulna distal to the end of the olecranon process with an associated dislocation of the radiocapitellar joint in combination with a radial head fracture alone or combined with a fracture of the coronoid process. All 10 patients met these criteria and their injuries were classified according to the systems of Bado and Jupiter et al. [4], [5]. Radial head fractures were classified according to Mason [8], fractures of the coronoid process according to Regan and Morrey [9], respectively. Demographics, injury patterns, and details of the surgical treatment were extracted from chart review. All 10 patients were available for follow-up examinations at an average of 12.3 months (range: 11–13 months) after the trauma. All patients were evaluated by the same experienced examiner blinded to the classification of injury.

The average age was 52.4 years (range, 18–83 years). Six women and four men were included. In four patients the injury resulted from a motor-vehicle accident, in three from a casual fall from a standing height. The remaining three fractures were caused by a fall from a height of at least four meters. In four of the patients, the injury was part of a polytrauma including additional skeletal lesions in three, thoracic or abdominal trauma in two, and head injuries in two patients. The dominant arm was affected in eight patients. According to the classification of Bado two type I and eight type II fractures were seen. Two open fractures were classified grade I according to the classification of Gustilo and Anderson [10]. No patient had associated neurologic involvement. All eight Monteggia fractures with posterior dislocation of the radial head were further classified according to Jupiter et al. into two type IIa, four type IIb, two type IIc and two type IId fractures. 

**Figure 1:**
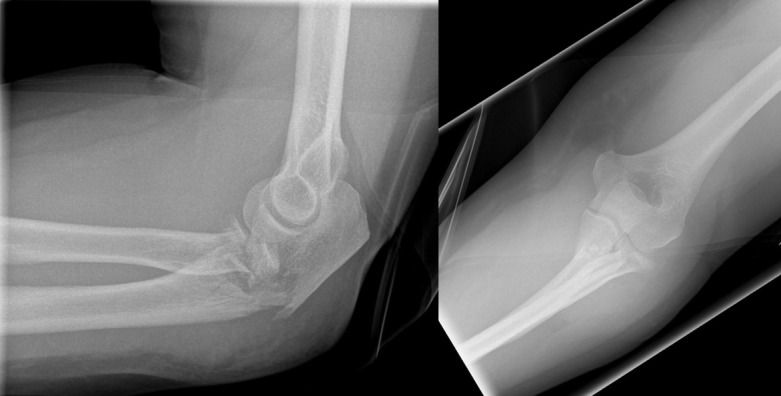
Male patient (43 years) with a posterior Monteggia-like lesion (Bado type II, Jupiter type IIB), Mason type III fracture of the radial head, and Broberg and Morrey type III coronoid fracture. Radiographs on the day of the injury.

**Figure 2:**
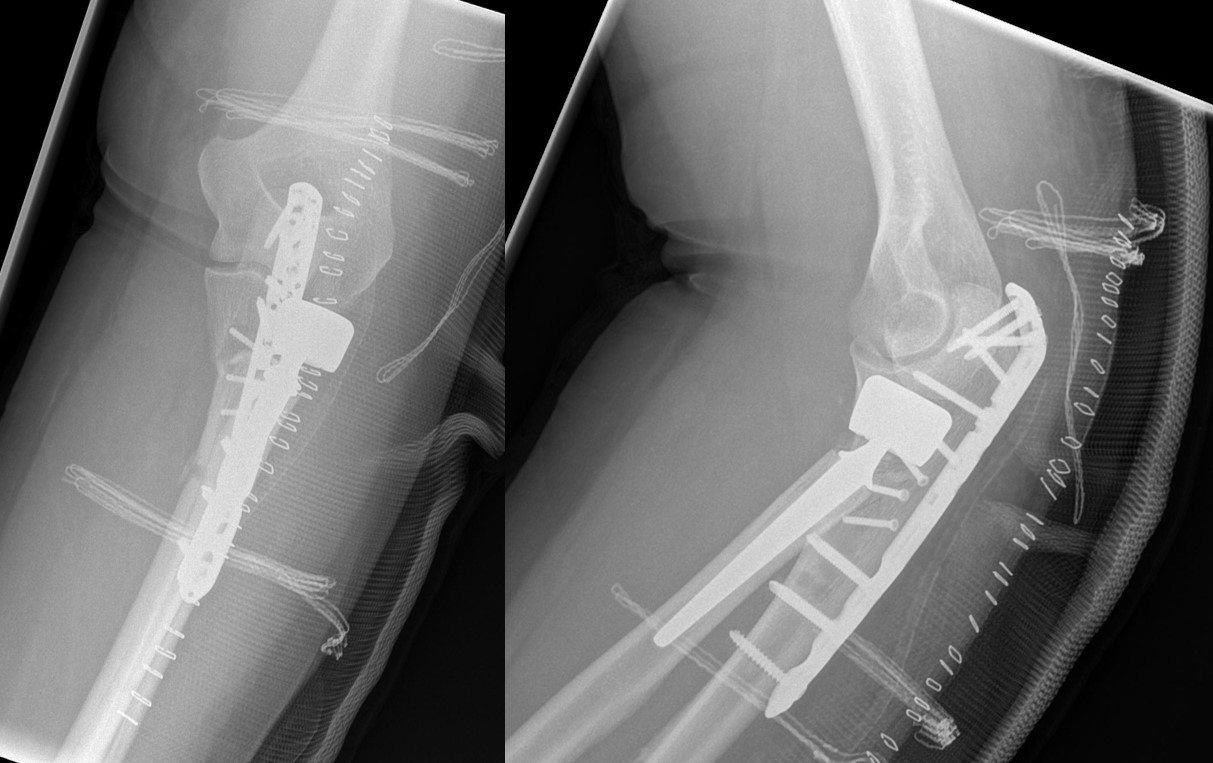
Postoperative radiographs. Osteosynthesis of the ulna was performed using a proximally precontoured olecranon plate applied to the posterior surface of the ulna. Implantation of a cemented bipolar radial head prosthesis.

**Figure 3:**
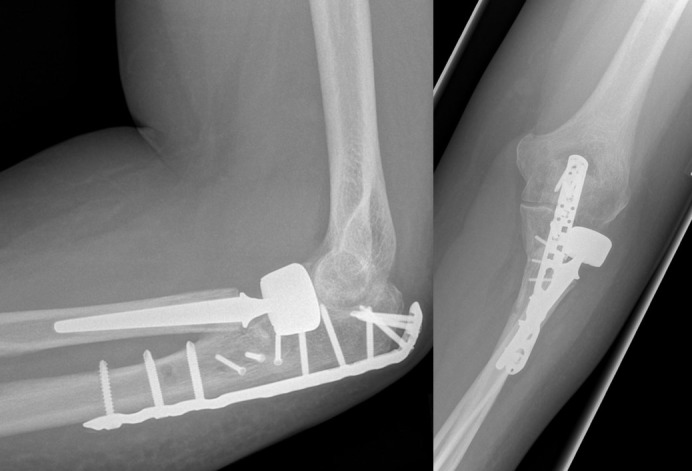
Same patient 11 months after the injury: no degenerative changes and heterotopic ossifications. No signs of loosening of the radial head prosthesis. Good clinical outcome.

Osteosynthesis of the ulna was performed using a proximally contoured 3.5 mm LCP (locking compression plate) or precontoured 3.5 mm LCP Olecranon Plate (both Synthes GmbH, Umkirch, Germany) applied to the posterior surface of the ulna in dynamic compression mode (Figure 1 (Fig. 1), Figure 2 (Fig. 2), Figure 3 (Fig. 3)). 

All patients had a fracture of the radial head. A total of five were comminuted (Mason type III), three were classified as Mason type II and two as Mason type I. All patients with a type III radial head fracture received a cemented bipolar radial head prosthesis (CRFII, Tornier, Montbonnot, France). All type II radial head fractures were treated with open reduction and internal fixation using mini screws. Type I fractures were treated with closed reduction of the radial head dislocation under fluoroscopic control. 

According to the Regan and Morrey classification five type I, three type II, and two type III coronoid fractures were included. All type II and type III coronoid fractures were associated with a Bado type II fracture and stabilized using lag screws inserted through the ulnar plate or with independent lag screws after indirect reduction of the fracture. 

All patients were immobilized in a long-arm cast for one week. However, immobilization was discontinued for daily immediate passive motion and continuous passive motion (CPM) without any restriction of movement starting at day 2 after surgery. Full weight bearing was allowed 6 weeks postoperatively. In order to prevent heterotopic ossifications non-steroid anti-inflammatories (Indomethacin 25 mg orally 3 times daily) were administered for 3 weeks. 

At the clinical follow-up examination the Mayo Modified Wrist Score (MMWS) [11] was used to assess the wrist function, while the Mayo Elbow Performance Score (MEPS) and the functional rating index of Broberg and Morrey [12] were used to assess the functioning of the elbow joint. The pain, satisfaction and stress resistance of the arm were recorded using the DASH Score as a subjective parameter [13]. The ROM of wrists, forearms and elbow joints was measured using a standard full-circle goniometer bilateral. The elbow was tested for valgus and varus instability in maximum extension and in 30° of flexion. In order to evaluate posterolateral rotatory instability, the pivot-shift test [14] was performed in each patient and stability was graded as normal, mild, moderate or severely unstable. Grip strength was measured with a Jamar dynamometer (Fabrication Enterprises Inc., White Plains, New York) with the other hand serving as a control. After three consecutive bilateral measurements, the grip strength at the injured side was expressed as a percentage of the control, using a correction factor of 1.07 for the dominant hand over the non-dominant [15], [16]. Patient satisfaction was determined by a subjective satisfaction questionnaire according to Jungbluth et al. [15]. Pain at rest and during activity was measured with a visual analogue scale (VAS).

Anteroposterior and lateral X-rays of the affected elbow joint were performed in all patients at the follow-up examination. Radiographs of the elbow were reviewed for capitellar osteopenia, degenerative changes, and heterotopic ossifications by an experienced and blinded radiologist. Capitellar osteopenia was graded as none, mild, moderate or severe according to Lamas et al. [17]. The degree of degenerative changes was classified according to Broberg and Morrey, as grade 0 (normal joint), grade 1 (slight joint space narrowing and minimum osteophyte formation), grade 2 (moderate joint space narrowing and moderate osteophyte formation), or grade 3 (severe degenerative changes with gross destruction of the joint) [12], [17]. Heterotopic ossification were graded as I, II, III or IV according to Brooker et al. [18]. In radial head prosthesis radiographic signs of loosening were assessed. Moreover, fracture union was defined as bridging bone on anteroposterior and lateral radiographs. 

## Results

Clinical follow-up examinations were performed at an average of 12.3 months (range 11–13) after surgery. With an average point value of 86.5 (55–100) for the MMWS good results were obtained. An average of 89.2 points (75–100) was registered for the MEPS and 20.1 (10.8–55.8) for the DASH score, indicating good results. The average point value for the functional rating index of Broberg and Morrey was 86.5 (57–100) indicative for a good result as well.

For forearm pronation mean values of 85° (70° to 90°) and for supination mean values of 75° (45° to 90°) were seen. Elbow flexion was from a mean of 9° fixed flexion (0° to 30°) to 131° (100° to 140°). Range of motion of the wrist displayed a mean palmar flexion of 75° (60° to 90°) and dorsal extension of 69° (40° to 90°). 

After application of the corrective factor for the non-dominant hand the grip strength was 91.8% (86% to 99%) of the contralateral side on average. No patient exhibited any wrist or elbow instability. The Pivot shift was graded as normal in all patients. 

According to the questionnaire for subjective patient satisfaction eight of the ten patients were satisfied with the results of the treatment and would choose the treatment regimen again. Eight patients reported no relevant restrictions of movement. All patients were able to return to their previous workplace and reached the same level of athletic activities as before the accident. The average VAS value was 1.0 (0 to 1) at rest and 1.7 (0 to 3) during activities. 

Eight patients exhibited neither signs of capitellar osteopenia nor degenerative changes or heterotopic ossifications in the region of the elbow on conventional radiographs. Two patients with a radial head prosthesis showed mild osteopenia and grade 1 degenerative changes. These patients exhibited grade 1 heterotopic ossifications. No patient required revision surgery. None of the patients showed signs of loosening of the radial head prosthesis. Fracture union was achieved in all patients.

## Discussion

Monteggia-like lesions are rare injuries and recommendations of treatment strategies are sparse. These injuries still represent a challenge to orthopedic surgeons. This challenge is further complicated since most studies about Monteggia injuries do not define Monteggia-like lesions as separate clinical entities [19]. Moreover, many studies on Monteggia injuries were carried out before the wide application of locking plates and some reports even have presented results of Monteggia injuries in children and adults together [4], [20], [21], [22]. Despite a better understanding of the biomechanical principles and advances in surgical treatment options, Monteggia injuries are still frequently associated with complications, poor functional outcomes, and high rates of revision surgery [23]. Furthermore, Monteggia injuries with associated radial head or neck fractures tend to have even worse outcomes [5], [19], [21], [24]. Givon et al. presented a combined series of Monteggia and equivalent lesions treated during a 10 year period [21]. They concluded that equivalent lesions with associated radial head fractures had worse functional outcomes than other types. Ten complications occurred in nine adult patients treated operatively. However, Givon et al. did not use validated outcome tools for assessing clinical and functional results, the equivalent lesion were not specifically evaluated, classified, and treated as separate clinical entities. Furthermore, children and adults were assessed together. 

Ring et al. reported on 48 patients with Monteggia injuries followed up after 6.5 years on average. The vast majority (38 patients; 79%) had Bado type II injuries of whom 68% (26 patients) had an associated radial head fracture. Ten of these patients also had a fracture of the coronoid process as a single large fragment. Most of the patients with Bado type II fractures had good or excellent results, with 112 degrees of ulnohumeral motion and 126 degrees of forearm rotation. The average point value for the functional rating index of Broberg and Morrey was 85 points indicating a good result. All five patients with a poor result suffered an associated fracture of the radial head. Four of these patients also had a fracture of the coronoid process. According to Ring et al., among the patients with a Bado type II fracture, cases with a fracture of the radial head were more likely to have an unsatisfactory result. However, a statistical difference between the patients with and without radial head fracture could not be detected. Although good clinical results were found in the majority of patients with Bado type II fractures, 50% required revision surgery within 4 months of the index procedure.

Egol et al. retrospectively reviewed clinical and functional outcomes after surgical fixation of ipsilateral fractures of the proximal ulna with associated fractures of the radial head or neck, and/or radial head dislocation (Monteggia variant/Monteggia-like lesion) [19]. 20 patients were evaluated at a mean of 2.3 years. Egol et al. reported slightly worse results compared to the aforementioned study by Ring et al. with a mean Broberg and Morrey score of 79.1 and a mean DASH score of 64.1 (worse outcome than those of the general population). Nine of 20 (55%) patients had good or excellent scores compared to the 83% satisfactory results Ring et al. reported. In addition, all complications in their patients were associated with Bado type 2 fracture patterns. Furthermore, seven patients developed heterotopic ossification and 14 of 20 patients arthritic changes.

Strauss et al. evaluated 23 patients with a Bado type II Monteggia injury associated with a fracture of the head or neck of the radius [25]. Six of those had an accompanied posterior ulnohumeral dislocation at the time of injury. The 17 patients without ulnohumeral dislocation were assessed at a mean follow-up of 29 months. In these patients the mean elbow flexion was 127° and the loss of extension was 5° at follow-up. The mean pronation was 60° and supination 67°. The mean standardized DASH score was 23 and the mean Broberg-Morrey functional index score was 83. This accounted for six excellent, four good, six fair and one poor outcomes. Radiographic assessment of post-traumatic arthritis showed six patients with Broberg-Morrey grade 0, nine with grade 1 and two with grade 2 changes. Mild heterotopic ossification was present anteriorly in two patients.

Konrad et al. evaluated 27 Bado type II fractures as part of a population of 47 Monteggia injuries after a mean time of 8 years. In this series eleven patients suffered a radial head fracture, a fracture of a coronoid process, or a combination of both (Monteggia-like lesions) [23]. An intra-articular fracture of the radial head and a fracture of the coronoid process were correlated with a poor Broberg and Morrey Score. The authors concluded that Bado type II Monteggia fractures further classified as Jupiter type IIa fractures, are frequently associated with fractures of the radial head and the coronoid process. This injury pattern should be considered as negative prognostic factor for functional long-term outcome. Unfortunately, Konrad et al. did not report the score results of the evaluated Monteggia-like lesions separately. Therefore, a comparison to our results is hardly possible.

However, compared to all aforementioned studies, where most of the authors did not particularly differentiate between Monteggia injuries and Monteggia-like lesion, our results are considerably better. What are the reasons for the good to excellent outcome scores and the low number of complications in our study?

At first, all patients received a CT scan preoperatively to evaluate the severity of all existing concomitant injuries of the elbow joint. Hence, all radial head and coronoid fractures could be detected, classified, and addressed. Furthermore, all of our patients were treated according to the same distinct treatment strategy. This was not done in other studies persistently [21], [23], [24], [25]. According to Ring et al. the key treatment principle in Monteggia fractures is stable anatomic alignment of the ulna [24], [26]. In adults, this alignment is achieved with plate and screw fixation [24].

In contrast to all aforementioned studies all of our patients received osteosynthesis of the ulna using modern fixation techniques. In all cases a proximally contoured 3.5 mm LCP (locking compression plate) or precontoured 3.5 mm LCP Olecranon Plate was applied to the dorsal surface of the proximal ulna in dynamic compression mode. Since posterior tensile forces are encountered at the apex of the proximal end of the ulna with active motion, a plate applied to the lateral or medial surface of the ulna is mechanically inferior to a plate applied to the posterior surface of the ulna, which works as a tension band [24]. Ring et al. recommend fixation of the ulnar fracture with a stout plate, such as a 3.5-millimeter limited-contact dynamic compression plate, applied to the posterior surface of the ulna and contoured proximally to reach the tip of the olecranon [24]. Semitubular or one-third tubular plates as well as tension band-wire constructs seem to be not rigid or strong enough. The proximal contour allows to address the proximal fragment with more screws. The most proximal screws are oriented at 90 degrees to the more distal screws, creating a more stable construct [24], [26].

Until today, the question whether it is better to treat a severely comminuted fracture of the radial head associated with a Monteggia-like lesion with radial head excision, reconstruction or prosthetic replacement remains unanswered [19], [24].

In our case series a total of five radial head fractures were comminuted (Mason type III), while three were classified as Mason type II and two as Mason type I. All patients with a type III radial head fracture received a cemented bipolar radial head prosthesis. No patient showed signs of loosening of the radial head prosthesis at follow up. All type II radial head fractures were treated with open reduction and internal fixation using mini screws. In all cases of type I fracture the treatment of the radial head dislocation was closed reduction with verification under fluoroscopy. Egol et al. showed that patients with radial head reconstruction or resection surgery achieved similar elbow function [19]. However, Ring et al. found better results in patients who had resections rather than attempted internal fixation [24]. Reynders et al. recommended against early resection of the radial head to improve outcome [27]. Nevertheless, the radial head plays an important role as a secondary stabilizer of the elbow joint in the absence of the medial collateral ligament [25]. Preservation of the length of the radial column by fixation or replacement seems to be a mainstay in the treatment of these injuries [25]. Konrad et al. treated all Mason type II radial head fractures with open reduction and internal fixation, and showed good or excellent results. In the same study Mason type III fractures were treated by radial head resection or reconstruction with poor results. According to the authors, not using prosthetic replacements as a treatment option might have led to these poor results, as the preservation of radiocapitellar contact with a prosthesis or a reconstructed radial head might increase the stability of the ulnohumeral articulation [23]. Furthermore, radial head resection may not be advisable since this may cause a proximal migration of the radius as a result of the frequently associated lesion of the interosseous membrane [6]. 

Fractures of the coronoid process can lead to instability of the ulnohumeral joint [9], [23], [28]. Therefore, large fractures of the coronoid process should be reduced anatomically to restore the ulnohumeral articulation and minimize the risk of ulnohumeral arthritis [23], [24]. This can be achieved with interfragmentary compression screws inserted through the posterior surface of the ulna either through or adjacent to the plate [24]. According to the Morrey classification there were five type I, three type II, and two type III coronoid fractures in our case series. All coronoid fractures were associated with a Bado type II fracture and were stabilized with lag screws through the ulnar plate or with independent lag screws after indirect repositioning of the fracture. This could further account for the good results and the mild degenerative changes observed. Furthermore, patients received standardized postoperative treatment with early immediate continuous motion, which was not performed in other studies [19], [23], [24], [27]. 

## Conclusion

The Monteggia-like lesions described in this series have rarely been reported before. Our findings demonstrate that good or excellent short-term results can be realized if the injury is classified correctly and a standardized surgical treatment of all components of the injury is achieved. Further studies with larger patient populations and longer follow up periods are needed to evaluate the long-term effectiveness of this treatment concept.

## Notes

### Competing interests

The authors declare that they have no competing interests.
